# Case Report: A *de novo* Variant in *NALCN* Associated With CLIFAHDD Syndrome in a Chinese Infant

**DOI:** 10.3389/fped.2022.927392

**Published:** 2022-07-13

**Authors:** Zhenyu Liao, Yali Liu, Yimin Wang, Qin Lu, Yu Peng, Qingsong Liu

**Affiliations:** ^1^Neonatology Department of Hunan Children's Hospital, Changsha, China; ^2^Neonatology Department of Changsha Country Maternal and Child Health Care Hospital, Changsha, China; ^3^GeneMind Biosciences Company Limited, ShenZhen, China; ^4^College of Pharmacy, Xiangnan University, Chenzhou, China; ^5^GeneTalks Biotech Co., Ltd., Changsha, China; ^6^Pediatrics Research Institute of Hunan Province, Hunan Children's Hospital, Changsha, China; ^7^Department of Cardiothoracic Surgery, Hunan Children's Hospital, Changsha, China

**Keywords:** NALCN, sodium ion leak channel, CLIFAHDD, neurodevelopmental retardation, *de novo* mutation

## Abstract

**Background:**

The *NALCN* encodes a sodium ion leak channel that regulates nerve-resting conductance and excitability. *NALCN* variants are associated with two neurodevelopmental disorders, one is CLIFAHDD (autosomal dominant congenital contractures of the limbs and face, hypotonia, and developmental delay, OMIM #616266) and another is IHPRF (infantile hypotonia with psychomotor retardation, and characteristic facies 1, OMIM #615419).

**Case Presentation:**

In the current study, a Chinese infant that manifested abnormal facial features, adducted thumbs, and neurodevelopmental retardation was diagnosed with CLIFAHDD syndrome. A trio-based whole-exome sequencing revealed that the infant harbored a *de novo* variant of the *NALCN* gene (c.4300A>G, p.I1434V).

**Conclusions:**

Our findings further enriched the variant spectrum of the *NALCN* gene and may expand the clinical range of *NALCN*-related disorders.

## Background

The *NALCN* encodes a voltage-independent, non-selective cation channel (1, 2) expressed mainly in the central nervous system (CNS) (3–6). NALCN plays a significant role in mediating a background Na^+^ leak current (IL-NA) that determines the nerve-resting conductance and excitability of different types of cells (including neurons). For instance, NALCN forms a sodium channel complex with UNC80 and UNC79 (7) and is involved in many physiological processes, such as respiration (8), intestinal pacing (9), and circadian rhythm cycle regulation (10).

The *NALCN* variants were associated with various diseases, such as IHPRF (infantile hypotonia with psychomotor retardation and characteristic facies, OMIM #615419) (11), and CLIFAHDD (congenital contractures of the limbs and face, hypotonia, and developmental delay, OMIM #616266) (12, 13). CLIFAHDD syndrome was first reported by 10(12). It is characterized by congenital contractures of the limbs and face, hypotonia, and developmental delay inherited in an autosomal dominant manner (12, 14). To date, all the published *NALCN* variants of patients with CLIFAHDD have occurred *de novo* to our knowledge (12, 13). IHPRF is characterized by severe hypotonia, psychomotor retardation, and characteristic facies at birth, or early infancy inherited in an autosomal recessive manner (15). Both CLIFAHDD and IHPRF were neurodevelopmental diseases, although they shared some characteristics (e.g., developmental delay and hypotonia), they are otherwise distinct from one another. Their major differences are whether they have distal joint contracture and different inheritance patterns (12, 16, 17).

This study identified a novel *NALCN* variant (NM_052867.3, c.4300A>G) in a Chinese patient with CLIFAHDD. Our findings further enriched the variant spectrum of the *NALCN* gene and may expand the clinical range of *NALCN*-related disorders.

## Methods

### Whole-Exome Sequencing

The DNA was isolated from peripheral blood. The Novaseq 6000 platform (Illumina, San Diego, USA) with 150 bp pair-end reads was applied for sequencing. The general procedure for whole-exome sequencing (WES) was as follows: genomic DNA was sheared to a fragment size of around 150 bp and with blunt-end, followed by the addition of deoxyadenosine at the 3'ends of the fragments. The genomic DNA library was created by ligating adaptors to the ends of double DNA strands that enable sequencing. The library was amplified by PCR and then, hybridized into a pool of biotinylated oligo probes specific for exons. Streptavidin magnetic beads were used to capture DNA-probe hybrids. Raw image files were processed through CASAVA v1.82 for base calling and generating raw data with adequate consensus coding sequences (CCDS) coverages (93.93%-94.96%, for depth ≥ 20).

### Data Analysis

Burrows-Wheeler Aligner tool was used to map the sequencing reads to the human reference genome (hg19/GRCh37), and PCR duplicates were removed by Picard v1.57 (http://picard.sourceforge.net/). Variation annotation was conducted according to the American College of Medical Genetics and Genomics (ACMG) guidelines (18) and the Enliven^®^ Variants Annotation Interpretation System authorized by Berry Genomics. The parent-child relationship was identified by King software (v2.2.5) (19) to confirm the *de novo* to confirm the *de novo* origin of the variant.

### Sanger Sequencing

Sanger sequencing was used to validate the variant identified by WES. Primers were designed by the Primer3 program (https://primer3.ut.ee/).

## Results

### Clinical Data

A 3-day-old boy was admitted to the hospital for 24 h due to jaundice. At 38 weeks of natural pregnancy, he was born to a 30-year-old pregnant woman, G1P1. There was no abnormality except for the cord around the neck during the entire term. The baby weighed 3,100 g, the height was 47 cm and the occipito-frontal circumference was 34.5 cm at birth, Apgar scored ten at 1 min, and five at 10 min. His parents are non-consanguineous without a family history of genetic disease.

**Figure 1:**
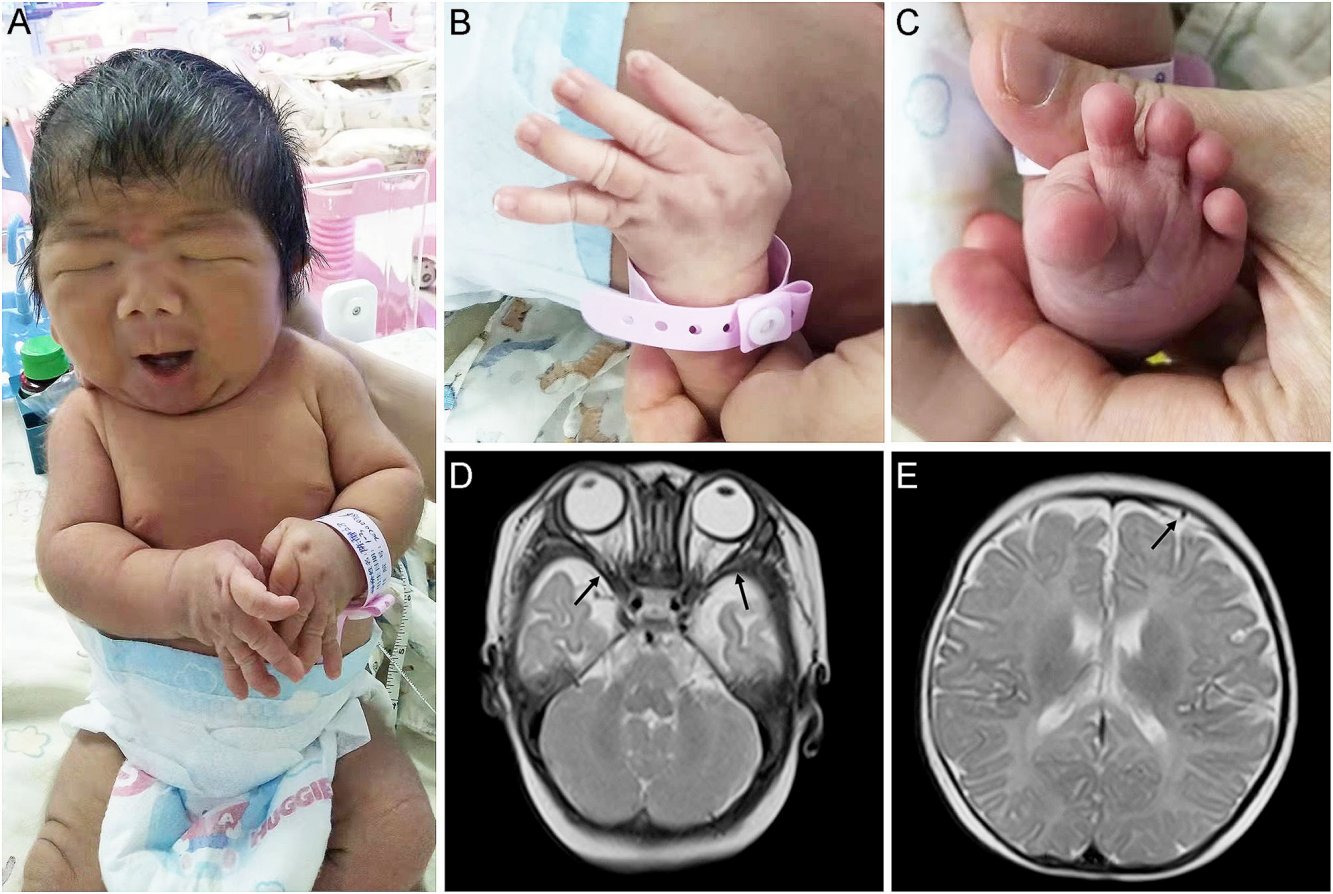
Appearance and MRI features of the proband. **(A,B)** Congenital contractures of the face **(A)** and limbs **(B)**. **(C)** Deformity of the foot. **(D)** abnormally broadening of bilateral temporal extracranial space. **(E)** Subdural effusion in the left frontal area.

Physical examination showed that the head, face, and trunk skin had moderate jaundice. Facial features included down slanting palpebral fissures, broad nasal bridge, large nares, long philtrum, micrognathia, pursed lips, H-shaped chin dimpling, deep nasolabial folds, and full cheeks were observed (Figure 1A). He also had an unusually short neck, adducted thumbs, clinodactyly of fingers, transverse palmar areas, elbow contracture, and toe deformity (Figures 1B,C). Anterior fontanelle (1.5^*^1.5 cm), pupillary morphology, and light sensitivity were normal. There was no apparent abnormality in the oral cavity, pharynx, lungs, chest, abdomen, anus, external genitalia, muscle tension of limbs, etc.

The MRI revealed that he has abnormally broadening bilateral temporal extracranial space (Figure 1D), left frontal subdural effusion (Figure 1E), and cyst in the right choroid plexus (3^*^2 mm). In addition, a color doppler ultrasound found that there was a patent foramen ovale in his heart (2.8 mm). His blood biochemical profile showed that he suffered from indirect hyperbilirubinemia, but the liver and kidney function was normal. Also, he suffered from anemia. After receiving phototherapy for jaundice, the infant's condition improved. Then, his parents decided to discharge him from the hospital.

Three months later, a physical examination showed that the infant manifested neurodevelopmental delay, unable to finish the Bailey Infant Development Scale. Moreover, his neonatal behavioral neurological assessment (NBNA) was only 31 points. The performance was observed as follows: no response of his head and eyes to the gurgling sound, the red ball, and the speaking face; incomplete support response at the upright position, grasp weakness, poor stretch reflection with only part of the body lifted, challenging to trigger the stepping reflex, and incomplete Moro reflex. At present, the proband is two years and six months old, and he is unable to speak his first words and he has no response to the orders with a score of 25 accessed by Gesell Developmental Schedules. However, he can still walk without fine motor control. His parents refused reexamination of the head by MRI and only took free rehabilitation training.

### Genetic Analysis

**Figure 2:**
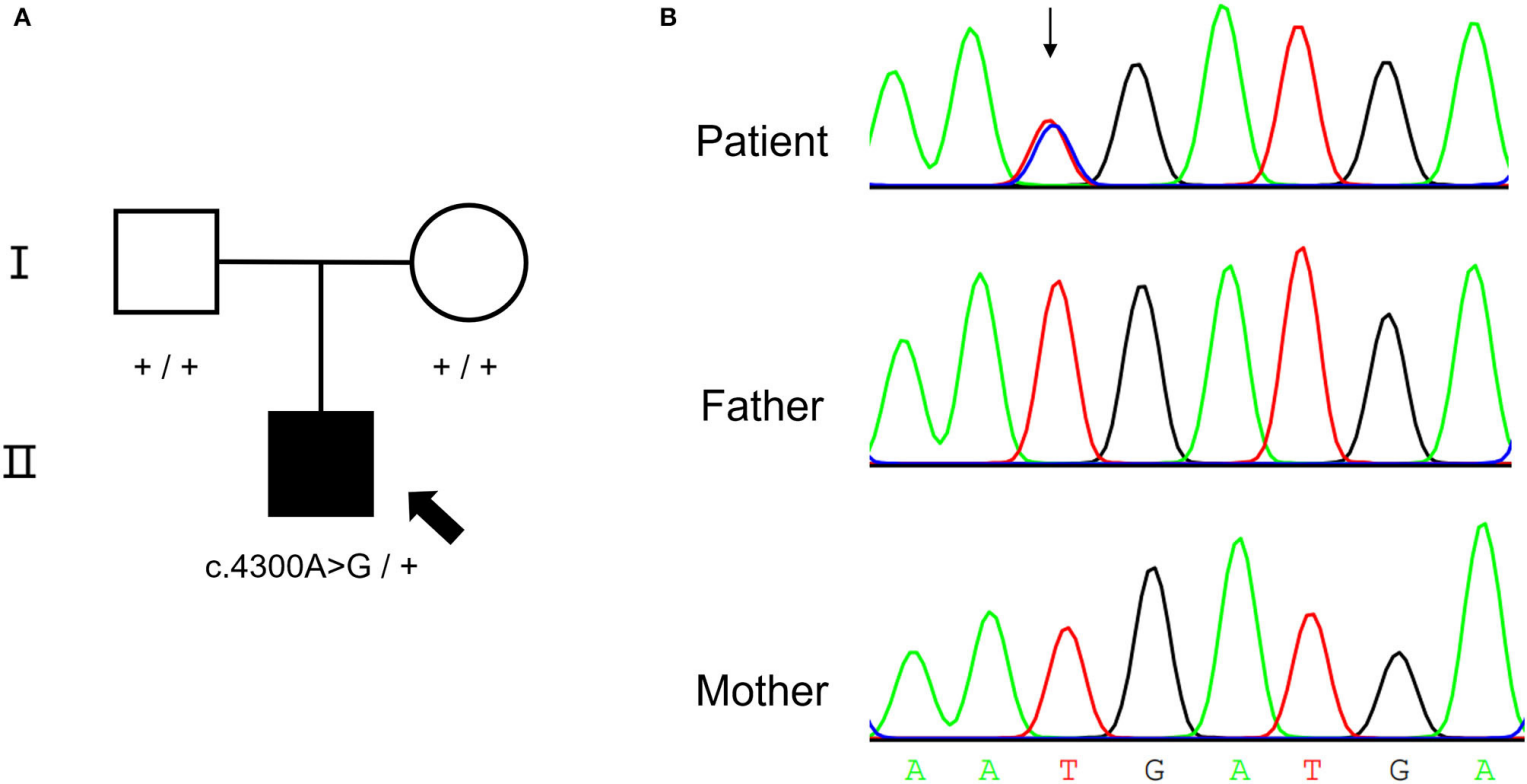
*De novo* NALCN variant of the proband. **(A)** The pedigree of the CLIFAHDD family. **(B)** Sanger sequencing result shows c.4300A>G in *NALCN* gene.

A *de novo* missense variant was identified in the *NALCN* gene (NM_052867.3: c.4300A>G, p.I1434V) by WES. The relationship between the three samples was confirmed (Supplementary Table S2). Sanger sequencing confirmed that the variant was heterozygous in the affected child but was absent from his parents (Figure 2). The variant was not found in gnomAD exomes or gnomAD genomes (http://gnomad.broadinstitute.org). In addition, the gnomAD missense Z score of the *NALCN* gene was 4.96 (> 3.09), and the assessment using multiple computational *in silico* tools indicated that the identified substitution was pathogenic or damaging (8 pathogenic vs. 3 benign predictions). Overall, the ACMG classification of the variant was likely pathogenic (PS2, PM2, PP2, and PP3).

## Discussion and Conclusions

The CLIFAHDD was first reported by Chong et al. (12). In this study, we reported a CLIFAHDD infant with a *NALCN* gene variant (c.4300A>G, p.I1434V). Besides, we found a cyst in the right choroid plexus (3^*^2 mm) in our patient, which was also found in the patient with the pathogenic variant p.Y578C (13). Cyst in the choroid plexus could be detected in 8.8% of neonates, and most of the cyst could be resolved spontaneously, the association between the cyst and developmental delay had not been built (20). At present, the status of the cyst in our case was not available, as the parents refused further examination.

The *NALCN* gene contains 1,738 amino acids and 44 exons, located on chromosome 13q33.1 (1). NALCN is a voltage-independent, non-selective cationic channel, which has four homologous repeats (I-IV), each composed of six transmembrane segments (S1–S6) (21, 22). The sodium leakage currents are transferred through the pore of the channel, four pore-forming loops (P-loops) spanning from S5 to S6 making up the ion selectivity filter, which is essential for controlling the resting and conducting membrane potential through persistent sodium leakage currents, contributing to nervous tension excitatory (21, 23). There were 23 *de novo NALCN* variants, which were reported to be associated with CLIFAHDD or distal arthrogryposis (6, 12–14, 16, 24–26), all were located in or near the S5 and S6 segments, and the clinical spectrum was reviewed (Supplementary Table S1). Previous reports indicated that facial contractures, arthrogryposis, joint contractures, hypotonia, and development delay (including cognitive delay, motor delay, and speech delay) were among the most prevalent manifestations; cerebellar atrophy was the most common abnormality found by MRI; although other clinical features varied. There were two previously reported variants (p.I1445L and p.I1446M) located in the S6 of the fourth domain, the patient with the p.I1445L variant presented no arthrogryposis and joint contractures (26), while the patient with p.I1446M variant presented no cerebellar atrophy (12). In our case, the proband displayed no hypotonia but suffered from facial contractures, arthrogryposis, and development delay. Our findings supported the high variability of the clinical phenotype caused by *de novo NALCN* missense variants. To date, there were no efficient treatments for CLIFAHDD, except for symptomatic treatments, such as clonazepam for seizures (24), acetazolamide for episodic ataxia (16), and surgical procedures for arthrogryposis (27). A 2-aminoethoxydiphenyl borate and a flunarizine could rescue the hyperactive phenotype of C. elegans caused by *NALCN* gain-of-function variants or it might serve as a direct blocker of the channel (28). However, further validation was needed for clinical implementation.

Contrarily, biallelic loss-of-function variants of *NALCN* often led to the IHPRF syndrome (15, 17, 29). In addition, the parents of patients with IHPRF were carriers of *NALCN* variants but reportedly did not show any symptoms of CLIFAHDD syndrome (12). The phenomena suggested that CLIFAHDD and IHPRF are caused by different mechanisms. Research has shown that some of these variants resulted in a dominant gain-of-function defect. For instance, four human CLIFAHDD variants (p.T513N, p.F512V, p.L509S, and p.L590F) in the orthologous position of the *C. elegans* result in gain-of-function phenotypes, which were previously proven (30), like hypertonia, smaller body size, and curly posture (24). Another research revealed that NALCN channels contribute to an inward Na^+^ background current in differentiated NG108-15 cells, and the current amplitude was a time-dependent decay; expression of the two CLIFAHDD variants, p.L509S, and p.Y578S, showed Na^+^ background current of significantly higher current density and a significant slowing of the current inactivation. On the contrary, expression of the IHPRF p.W1287L variant did not result in any detectable Na^+^ background current (31), indicating a non-functional NALCN channel. These results implied that gain-of-function variants of *NALCN* might result in CLIFAHDD, while loss-of-function variants might result in IHPRF. Further study was needed to explore the detailed mechanism.

Nevertheless, we also found a *de novo BPTF* variant (NM_182641.3: c.1117A>G, p.T373A) in this infant. It was classified as a VUS variant according to ACMG criteria (PS2_surpporting+PM2_supporting). A heterozygous variant of *BPTF* may cause neurodevelopmental disorder with dysmorphic facies and distal limb anomalies (NEDDFL). However, the NEDDFL manifested with abnormalities of the hands and feet, and the facial features are distinct from CLIFAHDD. Thus, we considered that the *BPTF* variant was not the causal variant.

## Conclusion

In conclusion, we first described CLIFAHDD phenotypes associated with a variant of the *NALCN* gene (c.4300A>G, p.I1434V) in a Chinese infant. Our findings further enriched the variant spectrum of the *NALCN* gene and may expand the clinical range of *NALCN*-related disorders.

## Data Availability Statement

The datasets presented in this study can be found in online repositories. The names of the repository/repositories and accession number(s) can be found below: https://www.ncbi.nlm.nih.gov/genbank/, 2565796.

## Ethics Statement

The studies involving human participants were reviewed and approved by the Ethics Committee of Hunan Children's Hospital. Written informed consent to participate in this study was provided by the participants' legal guardian/next of kin. Written informed consent was obtained from the individual(s), and minor(s)' legal guardian/next of kin, for the publication of any potentially identifiable images or data included in this article.

## Author Contributions

ZL, YL, and QLi: supervision, resources acquisition, and original manuscript writing and editing. ZL, QLu, and YW: methodology and genetic experiments. YP: data analysis and manuscript review and editing. All authors read and approved the final manuscript.

## Funding

This work was supported by a grant from Major Scientific and Technological Projects of Hunan Province for collaborative prevention and control of birth defects: 2019SK1014.

## Conflict of Interest

YW was employed by GeneMind Biosciences Company Limited, ShenZhen, China. QLu was employed by GeneTalks Biotech Co., Ltd. The remaining authors declare that the research was conducted in the absence of any commercial or financial relationships that could be construed as a potential conflict of interest.

## Publisher's Note

All claims expressed in this article are solely those of the authors and do not necessarily represent those of their affiliated organizations, or those of the publisher, the editors and the reviewers. Any product that may be evaluated in this article, or claim that may be made by its manufacturer, is not guaranteed or endorsed by the publisher.

## Supplementary Material

Supplementary Table S1 The previous reported *NALCN* variants and phenotypes in CLIFAHDD.

Supplementary Table S2 Relationship confirm by King software.
