# Supplementary material for: Case Report: A de novo Variant in NALCN Associated With CLIFAHDD Syndrome in a Chinese Infant
Source: Front Pediatr. 2022 Jul 13;10:927392. doi: 10.3389/fped.2022.927392 (PMC9326163; doi:10.3389/fped.2022.927392)
Supplement: Supplementary Table S2 — Relationship confirm by King software. [file Table_2.DOCX]

**Table S2 Relationship confirm by King software**

| ID1 | ID2 | Kingship* | Relationship |
| --- | --- | --- | --- |
| Proband | Father | 0.2391 | 1st-degree |
| Proband | Mother | 0.2334 | 1st-degree |
| Mother | Father | 0.0176 | Unrelated |

*An estimated kinship coefficient range >0.354, (0.177, 0.354), (0.0884, 0.177) and (0.0442, 0.0884) corresponds to duplicate/MZ twin, 1st-degree, 2nd-degree, and 3rd-degree relationships respectively (https://www.kingrelatedness.com/).
